# The link between electricity consumption and stock market during the pandemic in Türkiye: a novel high-frequency approach

**DOI:** 10.1007/s11356-024-32155-x

**Published:** 2024-02-10

**Authors:** Ömer Tuğsal Doruk

**Affiliations:** 1 Adana Alparslan Türkeş Science and Technology University, Adana, Turkey; 2 GLO, Essen, Germany

**Keywords:** COVID-19, Electricity consumption, Stock markets, Turkish economy, Spillover index, Time-varying vector autoregression, Time-varying Granger causality

## Abstract

This article examines the relationship between electricity consumption and the stock market in the Turkish economy during the COVID-19 pandemic. A novel high-frequency model is used, incorporating the hourly energy consumption and Borsa Istanbul (BIST) National stock market index variables. To determine the effect of electricity consumption on the stock market index and vice versa, a high-frequency VAR-based spillover approach, time-varying Granger causality, and time-varying Bayesian VAR analysis are employed. The findings reveal a positive and weak relationship between electricity consumption and the stock market but it has a time-varying nature in an emerging market context in the post-COVID-19 period in the Turkish economy.

**Supplementary Information:**

The online version contains supplementary material available at 10.1007/s11356-024-32155-x.

## Introduction

The COVID-19 can be counted as novel pandemic disease, first seen in Hubei and spread worldwide in early 2020. It has a tremendous effect on the world economy. The IMF (2020) emphasizes that the effect of the COVID-19 on the global economy is even worse than the 2008 financial crisis. COVID-19 can generate a very fragile subject for emerging economies in this context. Massive portfolio outflows (around $55 billion in the first week of the pandemic) from emerging markets were seen in the early stage of COVID-19 (IIF 2021).

The main motivation of this study is to identify the link between the real sector and the financial sector during the pandemic period (which can be considered as an important business cycle period) for emerging markets. In this framework, the link between electricity consumption and stock market is investigated with high-frequency data. The information obtained from this study may provide important findings for the real sector-financial sector linkage under high uncertainty during business cycle periods in an emerging market context and may make significant contributions to business cycle theory and provide important clues for this linkage for other emerging markets.

It is precisely at this point that during business cycle periods, these business cycles offer both capacity utilization (because electricity consumption provides us with important findings on modern industrial production) and output tracking in real-time due to its non-storable feature (as stated by Da et al. 2017). However, during the pandemic period, the business cycle determination based on this electricity consumption can change instantaneously based on shutdowns or strict rules. In fact, the impact of this electricity consumption on the stock market should also be monitored through high-frequency data due to the rapid response of both the pandemic and the stock market during the pandemic period. Moreover, this instantaneous monitoring should be done with more reliable (or less deviated) data or information. Electricity consumption data is highly reliable as electricity markets are highly regulated and have extensive disclosure requirements and accurate measurement (Da et al. 2017). In fact, for this reason, data or variables based on electricity consumption are frequently used in the business cycle literature (Jorgenson and Grilisches 1967; Burnside et al., 1995, 1996; Comin and Gertler 2006; Doruk 2023). Therefore, the connection between the real sector and stock markets (or equity markets) in emerging markets is essential to investigate in the pandemic period. Moreover, the literature on pandemic-driven energy consumption is also expanding (Aktar et al., 2021; Bashir et al. 2022; Chofreh et al. 2021; Kanda and Kivimaa 2020; Klemeš et al. 2021; Li et al. 2023; Qarnain et al. 2021; Priya et al. 2021; Siksnelyte-Butkiene 2021; Zanocco et al. 2021; Zhang et al. 2021); however, as far as the author is aware, there is no study on the relationship between energy consumption and capital markets during the pandemic period.

In the present study, the analyses use high-frequency electricity consumption data to catch the hourly COVID-19 lockdown or stringency effect on economic activity.^1^ In this context, the effect of economic activity on the stock market can be more reliable and precise. Therefore, the effect of electricity consumption on the stock market is examined using the high-frequency dataset during the business cycle for an emerging market: Turkey. By doing so, a VAR-based Diebold and Yılmaz (2009) spillover analysis, time-varying Bayesian VAR analysis, and time-varying Granger causality analysis are utilized.

In this study, unlike the previous studies, a real sector-financial sector relationship that has been addressed in the literature, especially through hourly data, is examined during the pandemic period, which is considered as an important business cycle period. Moreover, we know that the COVID-19 has a different impact on the economic activity in the different pandemic stages. Therefore, hourly data can grasp the heterogeneity of the effect of COVID-19 on economic activity and financial markets. It can be stated that such a dataset and methodology are rarely used in the current literature.

The findings from the VAR analysis-based spillover index, time-varying Bayesian VAR, and time-varying Granger causality analysis shed light on the stock market and economic activity relationship in the pandemic period for an emerging market. The main finding is that, in the case of a pandemic, proves a positive but weak relationship between electricity consumption and the stock market in an emerging market context. In other words, the relationship between real sector and financial markets is weak vice versa during the pandemic in an emerging market.

In sum, to the best of the author’s knowledge, there is no study examining the impact of high-frequency electricity consumption—which is an important business cycle indicator—on the stock market during the pandemic period that also takes into account the high-frequency noisy structure of a business cycle. For the above-mentioned points, the contribution of this study to the literature is manifold.

The present paper is organized as follows. “Literature review” section reviews relevant literature on the electricity consumption-stock market nexus. “Data and methodology” section gives the information of the dataset and describes the methodology. “Estimation results and robustness checks” section presents the empirical results, robustness checks, and discussion on the findings from the analysis. “Discussion” section summarizes the main findings and conclusion.

## Literature review

Economic activity can be proxied as electricity consumption in the current literature, as Apergis and Payne (2014) emphasize. As Rathnayaka et al. (2018, p. 70) underline that “… the relationship between energy consumption and economic growth has impacted directly both individual’s standards of living and industrial enhancements….”^2^ The energy consumption can be counted as a good proxy of economic performance (see Apergis and Payne 2009; Comin and Gertler 2006; Da et al. 2017; Dagher and Yacoubian 2012; Shahbaz et al. 2017). Figure C1 also shows the high relationship between electricity consumption and industrial production for the Turkish economy (the correlation between these series is more than 70%).

In the current literature, the effect of energy prices on the stock returns is examined (see Sadorsky 2001; Henriques and Sadorsky 2008; Park and Ratti 2008; Oberndorfer 2009; Jones and Kaul 1996; Gjerde and Saettem 1999).^3^ In their study, Apergis and Payne (2014) use electricity consumption as a proxy or indicator of economic activity for the OECD countries. Ferguson et al. (2000) emphasize that electricity is essential for economic growth, development, and sectoral production. Apergis and Payne (2014) note that electricity consumption is part of overall consumption in the economy, and therefore, it may play an essential role in asset pricing models. In the consumption risk-based approach, which was constructed by Campbell and Cochrane (1999), there is a very close link between macroeconomic variables and stock market behavior. In their simulation model, consumption growth significantly affects asset prices. Besides, Parker and Julliard (2005, p. 185) underline that, in their model, they use the consumption-based capital asset pricing model to examine whether its equilibrium risk to consumption determines the expected return of an asset. They find that consumption risk is an essential determinant of average returns across stocks. Yogo (2006) finds that the consumption risk for durable and nondurable has a different effect on asset prices.

Examining the effect of COVID-19 on the link between economic activity and the stock market can be counted as a novel in the current literature. The effect of COVID-19 on the stock market is also extensively researched on the fast-growing COVID-19 literature (for the most detailed COVID-19-related applied and theoretical papers, see Padhan and Prabheesh 2021, for the empirical studies, see Iyer and Simkins 2022). The impact of COVID-19 on energy consumption or energy markets is an important topic in the literature. For example, Aktar et al. (2021), Bashir et al. (2022), Chofreh et al. (2021), Krarti and Aldubyan (2021), Li et al. (2023), Mastropietro et al. (2020), Priya et al. (2021), Siksnelyte-Butkiene (2021), Zanocco et al. (2021), and Zhang et al. (2021) analyze the impact of COVID-19 on global or local energy consumption. Kanda and Kivimaa (2020) state that the COVID-19 pandemic could be an opportunity to transition to electric energy. Klemeš et al. (2021) examine the impact of vaccines on energy consumption during the pandemic. Qarnain et al. (2021) examine the impact of government policies to protect energy consumers. Sharifi et al. (2021) examine the impact of digital transformation on the energy sector during the pandemic. Moreover, Zhang et al. (2021) draw conclusions on what pandemic-focused energy literature or research methodologies should be.

There is also a business cycle literature as discussed in this study (Jorgenson and Grilisches 1967; Burnside et al., 1995, 1996; Comin and Gertler 2006; Doruk 2023). In this way, it contributes to this business cycle literature focused on electricity consumption within the framework of high-frequency real sector-financial sector connectedness during the business cycle.

All in all, according to the literature review, there is no study on the effect of COVID-19 on the link between electricity consumption and the stock market in an emerging market context. Therefore, the present study can fill an important gap in the current literature. The next part of the study presents the data, methodology, and empirical results.

## Data and methodology

### Dataset

The present study utilizes the hourly data on electricity consumption and stock market return. The time period covered by the sample of the study is considered as March 11, 2020 at 10:00 am and May 5, 2022 at 18:00 pm, following the announcement of the first official confirmed case on March 10, 2020, which is considered as the beginning of the pandemic (since the announcement of this first confirmed case coincided with the evening of March 10 and the first business day afterwards was selected). The hourly data are used for the electricity consumption data, and the data are taken from the EPIAS (2021) website (https://seffaflik.epias.com.tr/transparency/). The Borsa Istanbul National index (henceforth BIST National), the leading composite index for the stock market, is used for the stock market. The BIST National index hourly data are taken from Borsa Istanbul with the courtesy of Borsa Istanbul.^4^ The data consist of trading days, which do not have weekends. In the Turkish economy, the COVID-19-related lockdowns are applied during the weekend in the earlier phase of the pandemic. Therefore, the lockdown effect is eliminated by using trading days. Both variables are hourly basis. In order to guarantee the stationary, the differenced natural logarithm of series is taken. It also minimizes possible heteroscedasticity. Therefore, Delcons denotes the electricity consumption in its differenced natural logarithm, and DBIST denotes the differenced logarithmic value of the BIST National index.

Both variables are treated as logarithmic differences, i.e., growth rates. In this calculation, not only in order to eliminate unit root and heteroscedasticity problems, but also—and this is more important than the other reason—following Da et al. (2017), the growth rate of industrial electricity consumption can provide an important output gap measurement. Likewise, for the stock market, growth rate of stock market can provide us with an excess index development or vice versa. In this literature, small sample-based problems can also lead to regression-based overestimations (see Stambaugh 1999; Da et al. 2017). Table 1 summarizes the description of the variables, and Table A1 gives the descriptive statistics for the variables.

**Table 1:** Variable description

Variable	Description	The role of variable in the equation
DBIST	Difference between the logarithm of the current period value of the BIST National index and the logarithm of the previous period value	Dependent but endogenous high-frequency variable
Delcons	The difference between the logarithm of the current hour value of electricity consumption (MWh) and the logarithm of the previous hour value	Endogenous high-frequency variable

**Fig. 1:**
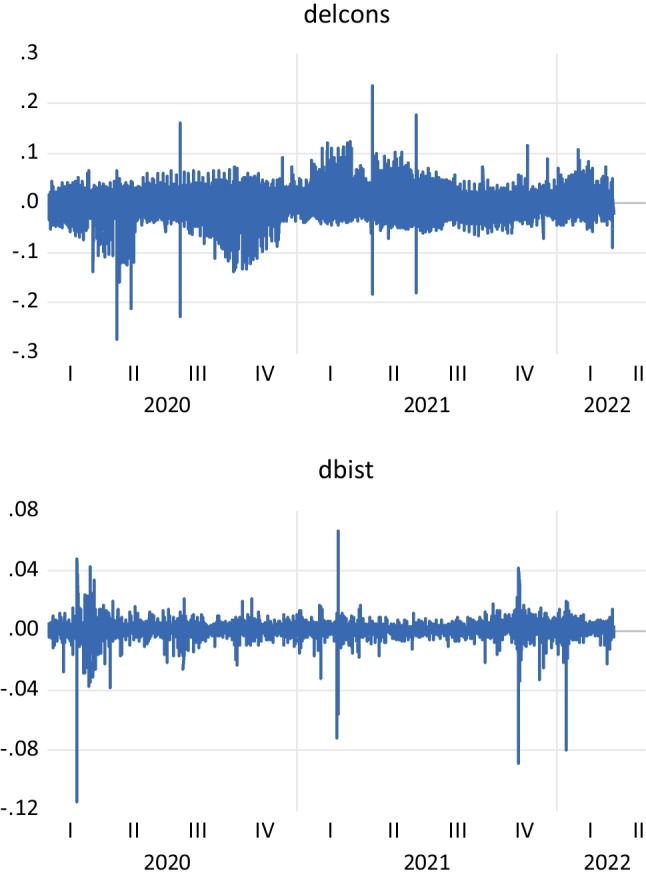
The electricity consumption and stock market (Borsa Istanbul) between 2018 and 2022 October, hourly, trading days

Table A1 presents descriptive statistics of the variables used in the study. The statistics in Table A1 show that these two variables are highly variable in the pandemic period. This is also seen from the minimum and maximum values of these variables. The number of observations is 5108. This number of observations is relatively high compared to the studies in the literature. Figure 1 is a graphical representation of these series. According to the results in Table A2 in the Appendix, the correlation between the two variables is quite low and negative during the pandemic period. A priori, this implies that the relationship between electricity consumption and equity market is quite weak and negative. However, in order to make final assessments, the analyses in the following sections of the study are carried out.

### Methodology

In this paper, the high-frequency VAR analysis-based Diebold and Yılmaz (2009) spillover index is used. The Diebold-Yilmaz spillover index is based on a simple VAR analysis. A simple VAR analysis can be expressed as in Eq. 1:

1 $${y}_{t}=\Theta (L){y}_{t}+{\varepsilon }_{t}$$

where *y*_*t*_ = {Dbist, Delcons}, and *L* is the polynomial matrix, and *ε* is the error term.

The spillover index is based on the forecast error variance decomposition, and then one-step ahead error vector can be written as follows:

2 $${\varepsilon }_{t+1,t}={y}_{t}-{y}_{t+1,t}={A}_{0}{u}_{t+1}=\left[\begin{array}{c}{a}_{{{}_{0,}}_{11}}{a}_{{{}_{0,}}_{12}}\\ {a}_{{}_{0}{,}_{21}}{a}_{{}_{0}{,}_{22}}\end{array}\right]\left[\begin{array}{c}{u}_{{{}_{1,}}_{t+1}}\\ {u}_{{{}_{2,}}_{t+1}}\end{array}\right]$$

where *E*(*ε*_*t*+1,*t*_, *ε*′_*t*+1,*t*_) denotes the covariance matrix.

Using the forecast error variance decomposition and the spillover index can be written for *N* variable VAR with *p*th lag, for *H* step ahead forecast, as follows:

3 $$S=\frac{\sum \limits_{h=0}^{H-1}{a}^{2}{}_{{{}_{0}}_{,i,j}}\sum \limits_{i,j=1}^{N}{a}^{2}{}_{{h}_{,i,j}}}{\sum \limits_{h=0}^{H-1}trace({A}_{h}A{^{\prime}}_{h})}\times 100$$

Equation 3 is the spillover index that is based on Diebold and Yılmaz (2009).

This study, therefore, uses the DY spillover index as the main methodology. As Han et al. (2020, p. 2) indicate that “…it is well-known that electricity spot prices exhibit an entirely different, more ‘spiky’ and volatile behavior than futures prices.” At the same time, the VAR model-based structure of the DY index, unlike the standard VAR, allows it to be easily aggregated and the spillover to be determined in a time-based manner, and in this respect, the DY index is able to model both electricity consumption and the stock market, which are highly volatile during an important business cycle period such as a pandemic, in a very robust manner. This DY index shows time-paths of shock transmissions across economic system more accurately and also, as a rolling window-based analysis, the DY index is a flexible analysis that does not need to specify any breakpoint or scenario (Han et al. 2020, p. 2).

The DY spillover index is the preferred methodology for macroeconomic analysis and is widely applied in the literature. The main advantage of the DY spillover index is that it generates a spillover index (through a forecast variance error decomposition) using parameters based on the widely accepted VAR model (Fu and Qiao 2022). In the VAR model estimated for this DY index, the DY index is inferred based on a generalized VAR model, which in fact uses a network topology and provides a table (connectedness table) on the direction of the spillover (Fu and Qiao 2022). It is a very convenient method in this respect and is easier to interpret than the classical VAR model and allows for a more efficient interpretation of real world-based spillovers with higher explanatory power and expresses the intensity of spillovers. The DY spillover index is based on the forecast error variance decomposition of overall spillovers based on a high-frequency-based VAR model. At the same time, according to Papież et al. (2022), these DY spillover index calculations are based on a structural constant parameter and depend on the order of variables in the variance–covariance matrix. In a sense, the DY spillover index is an analysis that calculates the spillover from one variable to another in financial markets or between two analyzed variables in a very simple and reliable way. The DY spillover index is a methodology frequently used in the literature for the connectedness of financial markets, volatility spillovers, and spillovers between macroeconomic variables (see Collet and Ielpo 2018; Yarovaya et al. 2016; Adekoya et al. 2021; Liu and Gong 2020; Elsayed et al. 2021; Fu and Quiao 2022; Papież et al. 2022). The DY index is also widely used in the literature for energy and commodity markets (Fu and Quiao 2022; Zhang 2017; Zhang et al. 2019; Liu and Gong 2020; Han et al. 2020).

Despite many advantages of the DY index, in this study, the results of the analysis in this study are analyzed again with two different methodologies, namely, time-varying Bayesian VAR and Granger causality methodologies since the DY index has the disadvantage that the variance–covariance matrix of the DY index depends on the order of variables.

## Estimation results and robustness checks

In this section of the study, we present the results and the robustness checks of the analyses applied for the exploring of the real sector-financial sector linkage for the Turkish economy during the pandemic period.

**Fig. 2:**
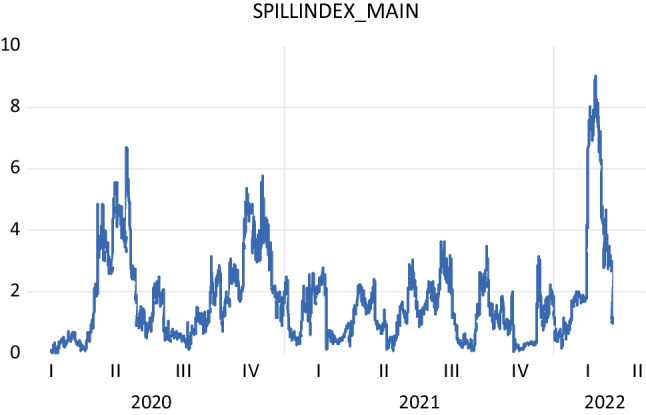
Diebold-Yılmaz spillover index results

A graphical representation of the calculated Diebold-Yilmaz spillover index is presented in Fig. 2. The Diebold-Yilmaz spillover index results in Fig. 2 are quite interesting. Especially in the COVID-19 period, the spillover between the electricity consumption and Borsa Istanbul is quite low. The obtained results show a positive relationship between electricity consumption and the stock market in an important emerging market: the Turkish economy, using the high-frequency electricity consumption and equity market information. In Table 2 and 3, the role of electricity consumption in the explanation of the Borsa Istanbul national index is quite low. The results obtained before the COVID-19 period are almost identical to those obtained during the COVID-19 period. The findings show that the dynamic relationship between electricity consumption and the stock or equity market is positive but quite low during and prior the pandemic. In other words, there exists a positive but quite low effect of electricity consumption on the BIST National index.^5^

**Table 2:** Spillover table for the pre-pandemic period

Spillover (connectedness) table			
	Delcons	DBIST	From others
Delcons	99.3	0.7	0.7
DBIST	0.0	100.0	0.0
Contribution to others	0.0	0.7	0.7
Contribution including own	99.4	100.6	0.3%

**Table 3:** Spillover table for COVID-19 period

Spillover (connectedness) table			
	Delcons	DBIST	From others
Delcons	100.0	0.0	0.0
DBIST	0.0	100.0	0.0
Contribution to others	0.0	0.0	0.0
Contribution including own	100.0	100.0	0.0%

We re-estimated the Diebold-Yilmaz spillover index during the COVID-19 period. It is found that there is a positive but very weak spillover from electricity consumption to equity market in the COVID-19 period. The findings obtained from this robustness check for the main model are almost similar in the COVID-19 period. These findings are presented in Table 3. These results suggest that the consumption-led stock market hypothesis is valid but the findings show that, again, such a relationship is very low in the Turkish economy in the pre- and post-pandemic periods. At the same time, the findings show that economic activity cannot stimulate the stock market index during the massive external shock, like the COVID-19. In contrast, the stock market cannot support economic activity. The results can be supported by the current literature that emphasizes that the stock markets cannot stimulate economic growth in the emerging markets since the low capital market deepening (see Levine and Zervos 1998) can be a vital link between the stock market and economic growth. During the pandemic, such a relationship still exists for the Turkish economy. The assumption of the high transmission from consumption to asset prices, which was advocated by Ferguson et al. (2000), Campbell and Cochrane (1999), Parker and Julliard (2005), and Yogo (2006), is not valid for the post-COVID-19 period for the Turkish economy.

It can be suggested that the uncertainty-related consumption shocks cannot significantly affect the stock market investment during the pandemic in the Turkish economy. In other words, economic activity and stock markets cannot be intertwined during the pandemic in the Turkish economy. As Goodell (2020) underlines that the COVID-19 can be counted as a black swan for the financial markets, the result can be reasonable in this manner.^6^

### Robustness checks

**Fig. 3:**
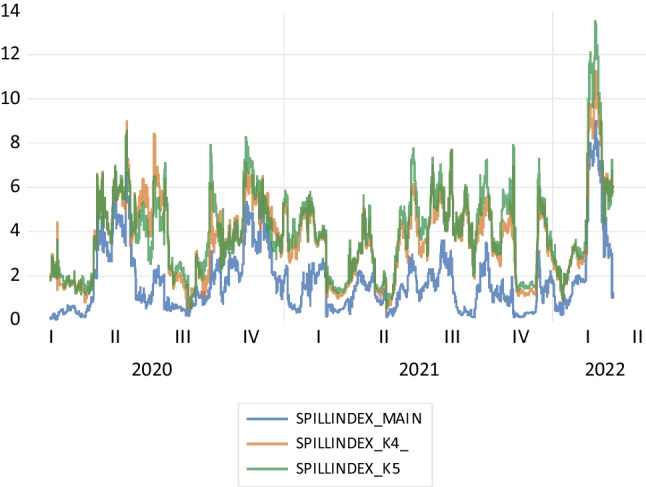
Different spillover index measurements

In this section of the present study, robustness checks are utilized to validate the findings of the main model. First, different lag levels are considered in this study. Different spillover indexes are estimated for randomly chosen lag levels and rolling windows. The findings are almost identical to the spillover index based on the main model. These findings are presented in Fig. 3 and Table 4.

**Table 4:** Different lags-based spillover index results

Spillover (connectedness) table			
	Delcons	DBIST	From others
Panel A. For lag length is taken as 4 (*k* = 4)			
Delcons	99.4	0.6	0.6
DBIST	0.1	99.9	0.1
Contribution to others	0.1	0.6	0.6
Contribution including own	99.5	100.5	0.3%
Panel B. For lag length is taken as 5 (*k* = 5)			
Delcons	99.6	0.4	0.4
DBIST	0.5	99.5	0.5
Contribution to others	0.5	0.4	0.9
Contribution including own	100.1	99.9	0.4%

### Time-varying Bayesian VAR model and Granger causality analysis

#### Time-varying Bayesian VAR model

In this section of the study, the time-varying Bayesian VAR model (TVP-VAR) is used to examine the time-varying hourly relationship between electricity consumption and stock market. In the TVP-VAR analysis, the Bayesian VAR parameters of Chan and Jeliazkov (2009) are used.^7^ However, robustness check of these parameters is also performed in the robustness check.

**Fig. 4:**
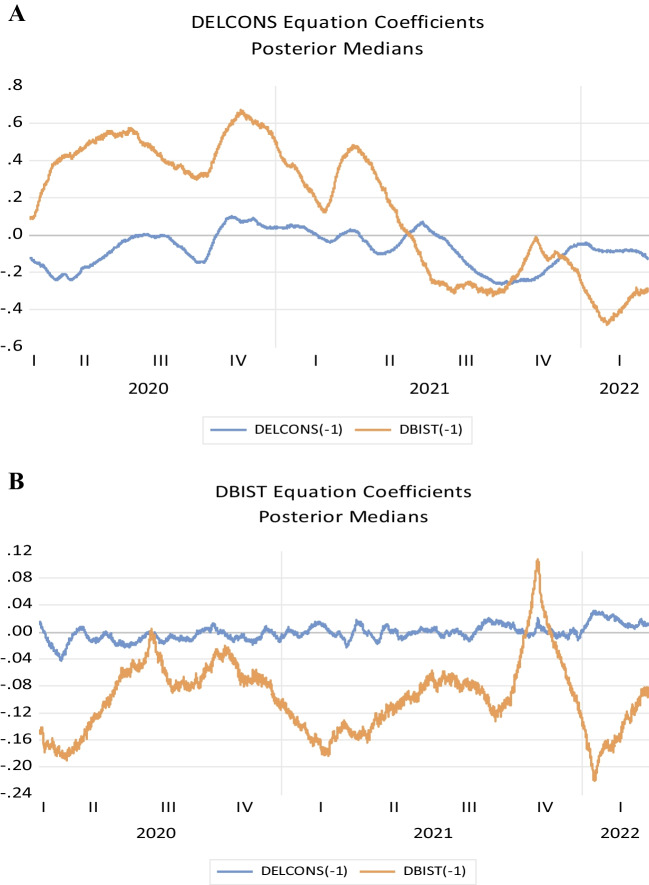
TVP-VAR model results

Figure 4 shows the results of the TVP-VAR model. It is observed that this relationship is decreasing in the first period of the pandemic, especially for the electricity consumption-stock market nexus, but then increases (see Fig. 4B). When Bayesian uncertainty is taken into account over time, the results show that this relationship has a high variation during the pandemic period. In parallel to the results found in the Diebold-Yilmaz spillover index in Fig. 4, the results of the analysis where the stock market (DBIST) is the dependent variable also show that the relationship between the stock market and electricity consumption is quite weak. The results in Fig. 4A are quite interesting. It is concluded that the stock market increased electricity consumption, or economic activity as it is considered in this study, in the first period of the pandemic.

#### Time-varying Granger causality test

The second time-based analysis method is the time-varying Granger causality test. Time-varying Granger causality test is based on the studies of Shi et al. (2018) and Shi et al. (2020) and it is observed that the Granger causality relationship changes over time. Granger causality underlines the fact that the direction of causality relationships changes over time, especially in macroeconomic and financial time series. For the Granger causality test, a time-based causality test is performed for the relationship between stock market and electricity consumption using a more general model with the recursive expanding Wald test.

**Fig. 5:**
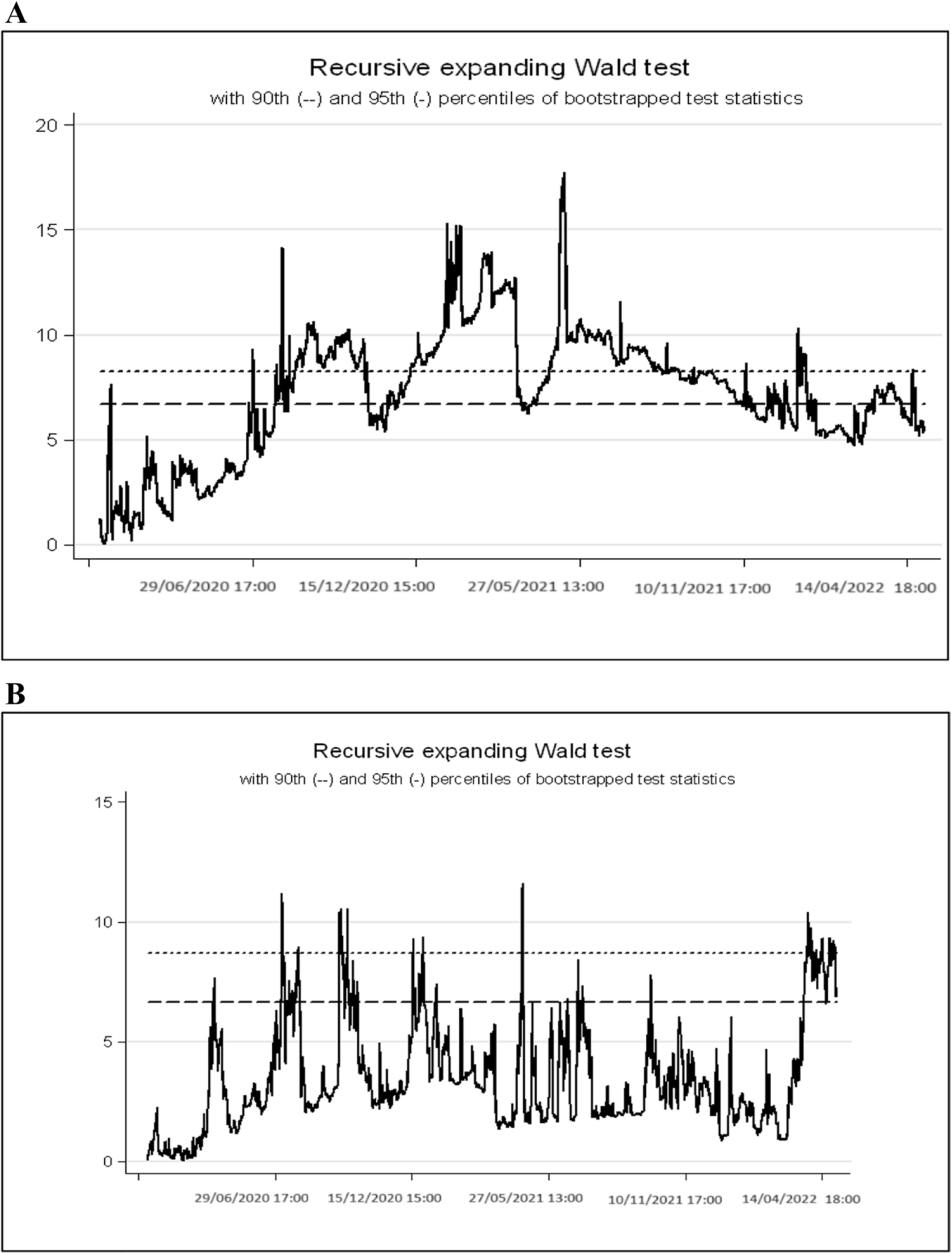
Time-varying Granger causality test results

Figure 5 shows the results of the time-based Granger test. According to these test results, stock market to electricity consumption causality is found to be valid in a small number of periods of the high-frequency sample according to the bootstrapped 95% significance level during the pandemic period. This relationship is also valid for electricity consumption to stock market. These results suggest that the high-frequency relationship between electricity consumption and stock market obtained through three different analyses in this study is rather weak.

### Extensions

#### Does the spillover between electricity consumption and stock market a martingale?

This part of the study investigates whether the spillover relationship between electricity consumption and the stock market is random or martingale. Martingale simply means random or not based on previous values. In other words, we test whether the tested spillover relationship is predictable or completely uncertain (random walk). According to the calculated Lo and MacKinlay (1988) test results in Table 5, the spillover relationship between electricity consumption and stock market is not martingale during the pandemic. In other words, this relationship is not a stochastic and uncertain relationship. In other words, the pass-through relationship between the stock market and electricity consumption can be predicted even if during the pandemic period. These findings take the validity of the consumption-based stock market approach in emerging markets one step further and show that this relationship is positive but weak and not stochastic.

**Table 5:** Martingale test results

Joint tests		Value	df	Probability
Max |*z*| (at period 7)		2.781862	4907	0.0060
Individual tests				
Period	Var. ratio	Std. error	*z*-Statistic	Probability
2	1.035759	0.020687	1.728568	0.0840
3	1.064157	0.030581	2.097958	0.0310
4	1.091510	0.037724	2.425765	0.0090
5	1.110741	0.043147	2.566591	0.0020
6	1.129509	0.047448	2.729517	0.0000
7	1.142806	0.051335	2.781862	0.0000
8	1.141436	0.055152	2.564464	0.0040
9	1.139587	0.058924	2.368944	0.0100
10	1.127176	0.062912	2.021489	0.0390
11	1.118934	0.067108	1.772262	0.0670
12	1.109899	0.071271	1.541987	0.1160
13	1.104085	0.075273	1.382775	0.1630
14	1.098636	0.079049	1.247786	0.2040
15	1.094801	0.082582	1.147956	0.2400
16	1.091713	0.085879	1.067933	0.2700

Test probabilities computed using wild bootstrap (1000 repetitions). Null hypothesis: SPILLINDEX_MAIN is a martingale. *N*: 4907 with heteroscedasticity robust standard error estimates

**Fig. 6:**
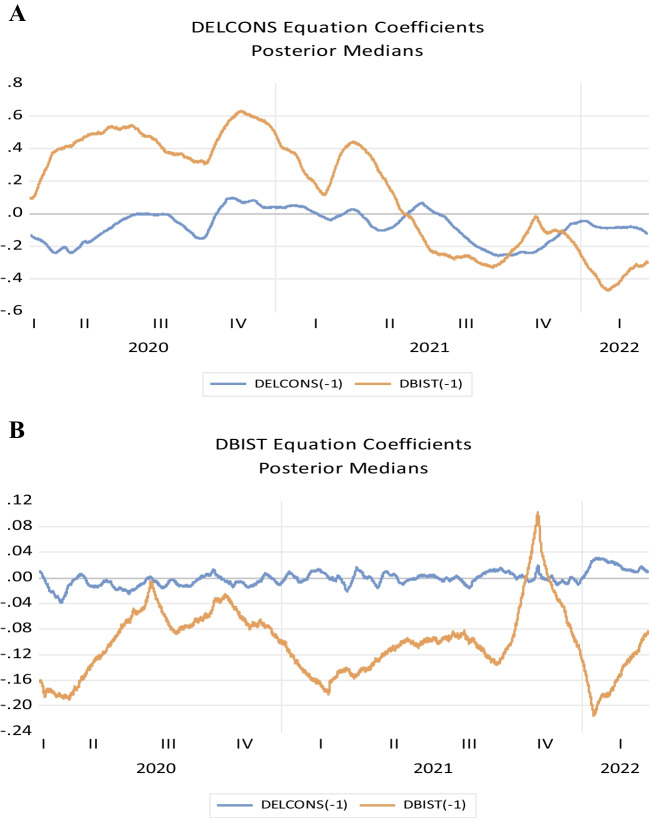
Results of TVP-VAR analysis

As a final extension, the TVP-VAR used in this study is estimated using different parameters other than the parameters of Chan and Jeliazkov (2009). In this extension, the TVP-VAR model was estimated using the standard parameters instead of the parameters of Chan and Jeliazkov (2009). The results obtained were found to be unchanged (Fig. 6).

All in all, the results of the time-varying analysis reveal some very interesting points. For the first of these points, when we look at the results of the time-varying VAR analysis using Bayesian uncertainty for the results of the analysis where the causality is from Borsa İstanbul to electricity consumption (Fig. 5A), it is seen that Borsa İstanbul supports electricity consumption or economic activity under uncertainty (this support is positive). However, it is observed that this relationship does not have a long-term structure afterwards. However, here we can see that the causality relationship from Borsa Istanbul to electricity consumption (especially when evaluated within the framework of dynamic structure) can be seen as important findings. Regarding reverse causality, when a causality relationship from electricity consumption to the stock market is examined, again, time-varying analysis (especially under both dynamic relationship and Bayesian uncertainty) expresses a different and time-varying causality relationship during the pandemic. The relationship from electricity consumption to the stock market is found to be quite weak during and after the pandemic period and slightly increasing after the pandemic (after this effect has passed).

When we look at the results of the TVP Granger causality analysis of this time-varying effect, these analysis results confirm both Diebold-Yılmaz and TVP-VAR Bayesian analysis results. The results of this Granger causality analysis, which underlines a relationship from electricity consumption to stock market at the beginning of the pandemic period, again express a causality, albeit weak, at the beginning of 2022. At the same time, it was found that the relationship from electricity consumption to stock market continued in a weak manner during the pandemic period.

## Discussion

This study examines the relationship between industrial electricity consumption growth, an important business cycle indicator that has been previously discussed in the literature, and the stock market. In this framework, three different time-varying methodologies that can reveal the power of high frequency are used. These methodologies are Diebold-Yilmaz spillover index, TVP-VAR analysis, and time-varying Granger causality analysis. All three methodologies show us that the relationship between electricity consumption (or more broadly, the business cycle indicator) and the stock market is quite weak during the pandemic period. In other words, during the pandemic period, when the business cycle is experienced with uncertainty, there is no increase in interest or orientation towards the stock market. These results of the study reveal different findings from the analyses of Da et al. (2017) and Kim et al. (2023), who investigate the electricity-stock market relationship in the literature. For example, Da et al. (2017) show that the relationship between each sector’s electricity consumption and the stock market is 10% (the *R*^2^ value of their model is around this value). They also note that sectors that are more prone to business cycles show this relationship to be more sensitive. This study was determined for the US economy and calculated on monthly electricity consumption. Kim et al. (2023) investigated the relationship between stock markets and electricity consumption for South Korea with monthly data. They find that there is a relationship between stock market and electricity consumption with extreme tail dependence using GARCH models. Moreover, this relationship was found to be tail dependence during the 2008 financial crisis. While both studies examine the relationship between stock market and electricity consumption, it is observed that this relationship is higher for the advanced economy than for the emerging market.

While investigating the relationship between electricity consumption and stock market, this study contributes to this literature in different ways. First, while both of these studies investigate the relationship between electricity consumption and stock market at the monthly level, this study investigates this relationship at the hourly level and with high frequency. At the same time, it is a study that investigates this relationship with different methodologies and reaches the same result by determining this relationship with three different analyses with time-varying characteristics. Another contribution is the study of stock market-electricity consumption for an emerging market by modeling a high-frequency, high-noisy dataset with significant uncertainty (hourly volatility was very important and necessary in financial markets during the pandemic). By accounting for high-frequency hourly correlations, this study finds that the relationship between electricity consumption and stock market is weak during periods of uncertainty, which is in line with the monthly findings of Kim et al. (2023). However, the results obtained in this study are similar to Kim et al. (2023) only in terms of findings.^8^

## Conclusion

The present study examines the effect of COVID-19 on the link between electricity consumption and the stock market index in the Turkish economy by using novel empirical approaches: the Diebold Yılmaz spillover index that based on the VAR analysis, Bayesian TVP-VAR analysis, and time-varying Granger causality analysis. A high-frequency hourly electricity consumption and stock market variables are used in the analysis; therefore, the dataset catches the pandemic-related economic and financial activities in the Turkish economy. During the earlier stage of the Turkish economy’s pandemic, stringent policies were taken to control human mobility to reduce the number of infected people in the Turkish economy; however, those policies are taken in weekends. Therefore, this effect is already absent in this dataset. This is a very rare data feature among the pandemic-related samples.

In the current literature, it is essential to explore the relationship between electricity consumption and stock markets during the business cycle. However, it has not been investigated straightforwardly using the high-frequency variables, especially for the emerging market-related literature for the pandemic period. Moreover, we know that the COVID-19 has a different impact on the economic activity in the different pandemic stages. Therefore, hourly statistics are able to comprehend the varied nature of COVID-19’s impact on financial markets and economic activities. It may be said that the existing literature does not often use a dataset and approach like this one. Therefore, it can be stated that the present study can contribute to the extant literature on the relationship between electricity consumption and the stock market, especially for an emerging market context. The obtained findings show that the link between electricity consumption and the stock market index is positive but very weak for the Turkish economy during the pandemic period. As such, the obtained findings shed new light on the interrelations between COVID-19, electricity consumption, and the stock market in an emerging market using hourly dataset. The present study is also to contribute to the COVID-19-related financial market literature. There is a limited but highly engaged literature on the economic impacts of the COVID-19 on the stock markets (see the reviews of Padhan and Prabheesh 2021, and Iyer and Simkins 2022).

According to the results of this study, some policy recommendations can be discussed. Among these policy recommendations, it is particularly important to note that the real sector-private sector relationship is quite weak but positive during business cycle periods. Important inferences can be made about the weak pass-through in this relationship. Since the decline in economic activity during business cycle periods is reflected in financial markets in a very short-term manner, it is suggested that financial markets should be examined and monitored separately by policymakers and any manipulation or financial speculation should be handled independently of economic activity. This is the most important policy implication of this study and it is based on the results of this study that financial instability should be treated independently of real activity during business cycle periods as an important policy implication for emerging markets.

There are some limitations that the present study faces. The dataset is only available for one country. Therefore, the obtained findings cannot be generalized to other countries, while the results can give important insight into the effect of COVID-19 on the developing and emerging market economies. Further studies may use the cross-country analysis. In addition, further studies may use detailed case studies for developing economies.

### Supplementary Information

Below is the link to the electronic supplementary material.

Supplementary file1 (DOCX 103 KB)

## Data Availability

It can be requested from the author upon reasonable request since the dataset is not permitted to distribute by the Borsa Istanbul Data Center.

## Footnotes

1 See Appendix Figure B1 and B2 for the pandemic-related information for the Turkish economy.

2 Since the work of Kraft and Kraft (1978), there has been vast literature on electricity consumption and economic growth. The link between electricity consumption and economic growth is underlined in the current literature (see Ozturk 2010 and Payne 2010a, b for the extensive surveys).

3 The effect of economic activity on the stock market is pointed out by Apergis and Payne (2014) for the first time in the current literature.

4 This data is made available to researchers by the courtesy of the Borsa Istanbul as high-frequency data by request. Therefore, we would like to thank Borsa Istanbul for making this study possible.

5 Lockdown policies implemented during the pandemic have almost no impact here. This is because curfews and restrictions in Turkey during the pandemic period were generally and mostly on weekends. Since trading days are used in this analysis, this effect is not included.

6 The effect of COVID-19 on the stock market volatility is also linked with the financial development level of the economy, as Uddin et al. (2021) emphasize. Before the pandemic, it is also underlined by Dellas and Hess (2005) and Uddin et al. (2021), the developed financial market has a well-structured capital market that efficiently allocates the funds across the parties within an economy. The capital market development can be counted as relatively poor in the Turkish economy. It is expected that there is a strictly positive and sensitive relationship between electricity consumption and the stock market in the Turkish economy during the pandemic.

7 In this analysis, a time-varying analysis is performed, taking into account parameter uncertainty due to pandemic-related uncertainty. I thank the anonymous referee for this TVP-VAR modeling suggestion.

8 It can be said that this study also has the potential to contribute significantly to the literature on the energy consumption-based asset pricing models and energy prices-stock market nexus. While the consumption-based asset pricing models and the energy prices-stock market nexus are popular in the current literature (see the works of Campbell and Cochrane 1999; Parker and Julliard 2005; Yogo 2006; Sadorsky 2001; Henriques and Sadorsky 2008; Park and Ratti 2008; Oberndorfer 2009; Jones and Kaul 1996; Gjerde and Saettem 1999), there is limited literature on the link between electricity consumption and stock market index in an emerging market context.

## Code availability

It can be requested from the author upon reasonable request.
