# Supplementary material for: The link between electricity consumption and stock market during the pandemic in Türkiye: a novel high-frequency approach
Source: Environ Sci Pollut Res Int. 2024 Feb 10;31(11):17311–23. doi: 10.1007/s11356-024-32155-x (PMC11289346; doi:10.1007/s11356-024-32155-x)
Supplement: Supplementary file 1 — Supplementary file1 (DOCX 103 KB) [file 11356_2024_32155_MOESM1_ESM.docx]

**Appendix**

**Appendix A. Additional Findings**

**Table A1. Descriptive Statistics**

| **Variable** | **Obs** | **Mean** | **Std. dev.** | **Min** | **Max** |
| --- | --- | --- | --- | --- | --- |
|  |  |  |  |  |  |
| **dbist** | 5,108 | .0001427 | .0058303 | -.1153393 | .0665688 |
| **delcons** | 5,108 | -.0000344 | .0300566 | -.2744312 | .2350349 |

**Table A2. Correlation Analysis**

| Included observations: 5108 | |
| --- | --- |
|  |  |
|  |  |
| Correlation | |
|  | DELCONS |
| DBIST | -0.01 |
|  | p-val=0.33 |
|  |  |
|  |  |

**Appendix B. Turkey in the COVID-19**


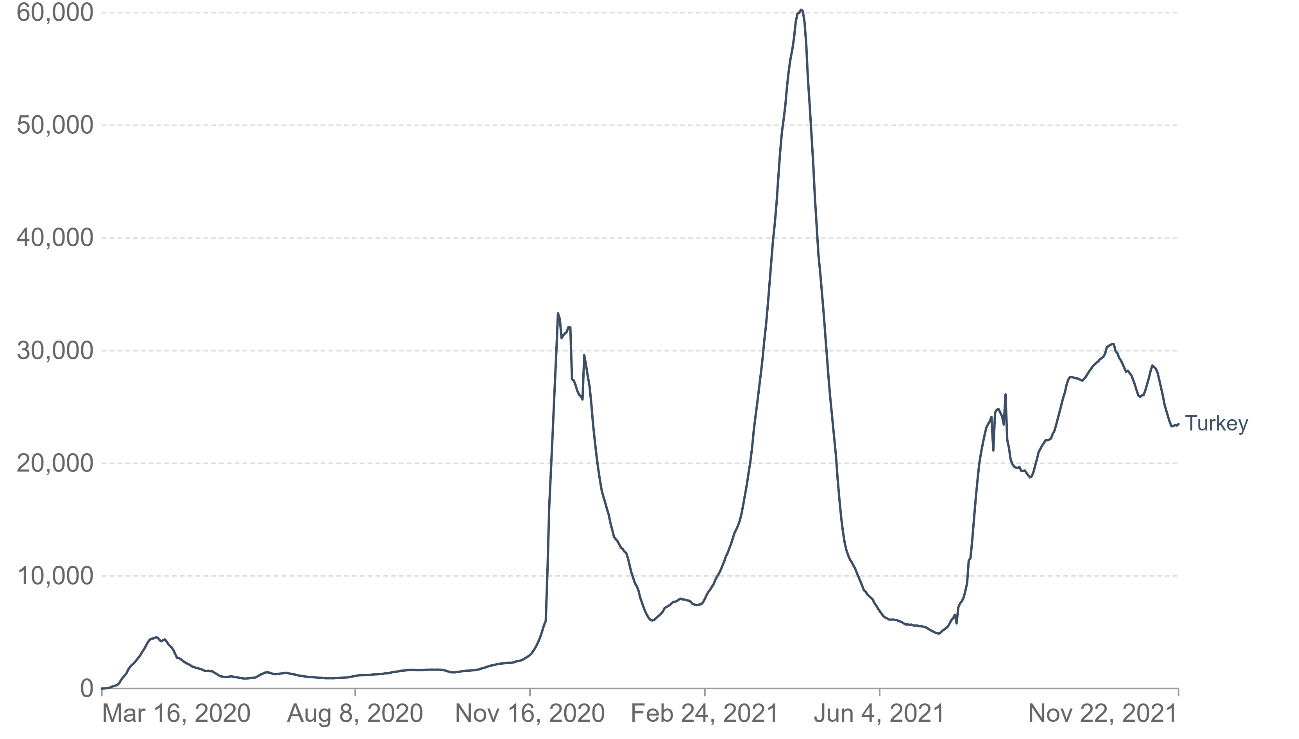


Source: Our World in Data, Oxford University, 2021

**Figure B1. The seven days rolling average of daily confirmed cases of COVID-19**

Source: Our World in Data, Oxford University, 2021

**Figure B2. Stringency Index during the COVID-19 period in Turkey**

**Appendix C. The Relationship between Electricity Consumption and Industrial Production**

**Source:** CBRT,2023 for industrial production index. Electr_cons: electricity consumption (monthly, averaged): the EXIST Database EPİAŞ (2023).

**Figure C1. The Relationship between Industrial Production, Capacity Utilization Ratio, and Electricity Consumption in the Turkish Economy in the pre-pandemic period, Monthly, 2019 January, 2020 December**
